# Editorial: Fostering self-regulated learning

**DOI:** 10.3389/fpsyg.2025.1681074

**Published:** 2025-10-29

**Authors:** Slavica Šimić Šašić, Orna Heaysman, Barbara Otto

**Affiliations:** ^1^Department of Teachers and Preschool Teachers Education, University of Zadar, Zadar, Croatia; ^2^Department of Education, Kaye Academic College of Education, Be'er Sheva, Israel; ^3^Faculty of Education and Leadership, Achva Academic College, Havat Shikmim, Israel; ^4^Fachhochschule der Diakonie, Bielefeld, Germany

**Keywords:** self-regulated learning (SRL), teaching practice, teacher education, instrument validation, direct instruction

In recent years, scientific research and literature have paid great attention to the ability of students to self-regulate their learning. Numerous theories and models of self-regulated learning (SRL) have been developed (Boekaerts, 2011; Butler and Cartier, 2018; Efklides, 2011; Hadwin et al., 2011; Pintrich, 2000; Winne and Hadwin, 1998; Zimmerman, 2000). Researchers agree that SRL is a cyclical, multidimensional process that includes the interaction of personal (cognitive, metacognitive, motivational, emotional), behavioral, and environmental factors (Panadero, 2017), which enable students to better manage their learning. The ability to self-regulate learning contributes to positive educational outcomes, but also to the development of lifelong learning skills, which facilitate coping with the demands of modern society. However, most students' learning is not optimally self-regulated (Kramarski and Michalsky, 2009). Teachers, as agents of SRL, can promote it in a variety of direct and indirect ways: by teaching students effective learning strategies or by structuring stimulating learning environments (Dignath-van Ewijk and Van der Werf, 2012). Most teachers agree that students need help to become self-regulated learners and express positive beliefs about SRL. However, they feel uncertain about how to promote students' SRL, thus they stimulate SRL to a limited extent (Dignath and Büttner, 2018; Dignath-van Ewijk and Van der Werf, 2012; Karlen et al., 2020; Kistner et al., 2015; Perry et al., 2008; Spruce and Bol, 2015; Šimić Šašic et al.´, 2023; Vandevelde et al., 2012). Teaching students how to self-regulate their learning improves their performance. However, it is still unclear which specific learning strategies should be taught and how they should be taught in order to improve student performance (Heaysman and Kramarski, 2022). Research also shows that the effectiveness of strategies varies according to discipline (reading, writing, mathematics, science; De Boer et al., 2012).

Encouraging SRL depends on numerous factors related to the teacher (teacher beliefs, gender, teaching experience, competences, etc.), class, school, subject, but also the students themselves (abilities, age, SES, etc.; Chatzistamatiou et al., 2013; De Smul et al., 2018; Dignath-van Ewijk, 2016; Dignath-van Ewijk and Van der Werf, 2012; Elmas et al., 2011; Fauzi and Widjajanti, 2018; Hargreaves, 2005; Karlen et al., 2023; Lombaerts et al., 2007, 2009; Moos and Ringdal, 2012; Peeters et al., 2015; Šimić Šašic et al.´, 2021, 2023; Vandevelde et al., 2012; Yan, 2018). Therefore, the results of studies on fostering SRL are often inconclusive.

## Overview of the Research Topic

Most studies on the promotion of SRL by teachers have been conducted on prospective teachers, as part of the evaluation of teacher training programmes to foster SRL; however, there is little research in the area of teachers' practice in promoting SRL. Therefore, the aim of this Research Topic was to gather new knowledge about the factors influencing the activation of SRL from the perspective of teachers, students, classes, schools, and even from the perspective of educational policies of different education systems (countries). Of particular interest was to examine the effects of different strategies for improving SRL, taking into account different characteristics of students, fields/disciplines, and levels of education (early childhood education, primary, secondary, or higher education), and to examine the mechanisms of these effects. It was necessary to investigate how students and teachers perceive the effectiveness of different methods of promoting SRL.

Therefore, this Research Topic aimed to offer a synthesis of the latest research on the promotion of SRL and to collect original contributions that can offer new insights and stimuli for future research. This Research Topic includes 18 theoretical and empirical, qualitative and quantitative articles and contributions from 68 authors. The research brings innovative concepts and methodology, applied in the classical classroom environment as well as in the online learning environment, from preschool to higher education levels, in different fields of learning, from different parts of the world.

**Figure 1:**
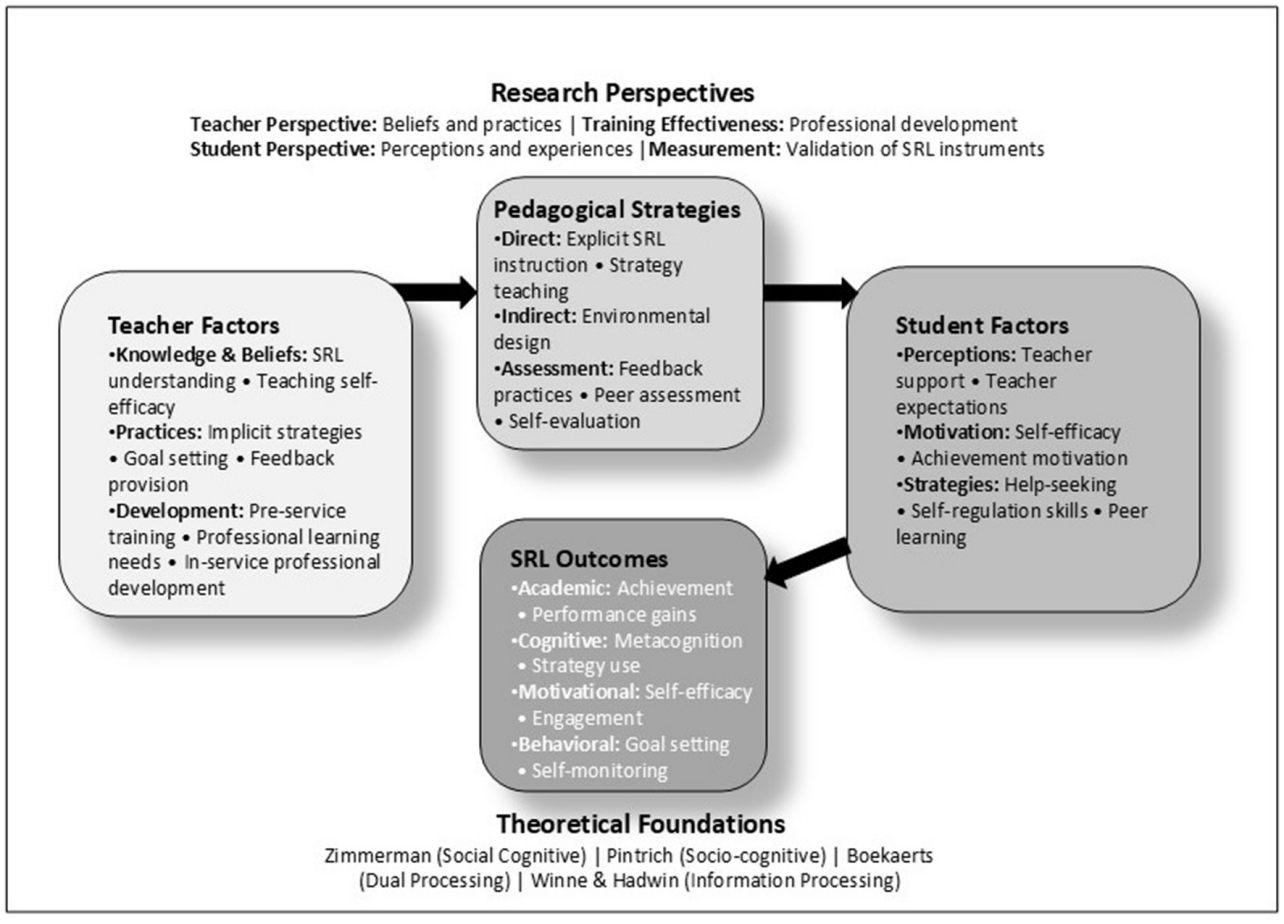
Fostering self-regulated learning: A conceptual model.

To interpret the diverse approaches to fostering SRL represented in this Research Topic, we draw on Dignath and Veenman's (2021) distinction between direct and indirect methods of promoting self-regulation. Direct approaches involve explicitly teaching SRL strategies to students (e.g., metacognitive training, strategy instruction), while indirect approaches create supportive environments and conditions that implicitly foster self-regulatory behaviors (e.g., through feedback systems, relationship-building, or classroom structures). Some combine both approaches. This framework helps illuminate patterns across our thematic categories. The papers can be divided into four categories: teacher perspective, training effectiveness, student perspective, and validation of measurement instruments (see Figure 1 for a visual summary of the Research Topic, and Table 1 for a detailed summary).

**Table 1:** A summary of the research topic papers.

**Authors (A–Z)**	**Short title of manuscript**	**Main topic/aim**	**Target group**	**Direct/indirect approach**	**Research methods**
1. Amir	Literacy resilience	Investigating “literacy resilience” as connecting literacy and SRL	Teachers	n/a	Quantitative
2. Backers and Van Keer	Professionalizing schools on self-regulated learning	Examining leaders' roles and responsibilities in guiding SRL-focused PD	School leaders	Combined	Qualitative
3. Barz et al.	Fostering SRL using digital learning environments	Analyzing pre-service teachers' SRL profiles in digital learning environments	Pre-service teachers	Comparing direct/indirect	Quantitative
4. Evans et al.	Developing a scale to explore self-regulatory approaches	Developing a scale about SR assessment and feedback (SRAF)	Higher ed. teachers	Indirect	Quantitative
5. Greenquist-Marlett et al.	Teachers' perceptions of their SRL practices	Investigating teachers' SRL strategies in K−5 contexts and how it is linked to self-efficacy and effectiveness	Teachers	N/a	Qualitative
6. Kumyoung	Development of a causal model of SRL	Examining a casual model of SRL	Higher ed. students	N/a	Quantitative
7. Manuel et al.	Encouraging self-regulated learning	Investigating how feedback promotes learners' SRL skills	Teachers	N/a	Qualitative
8. de Ruig et al.	Understanding the interplay	Identifying contextual determinants of students' SRL	Teachers	Combined	Mixed-method
9. Ortega-Ruipérez and Correa-Gorospe	Peer assessment to promote SRL with technology	Investigating how technology facilitates SRL using peer assessment activities	Higher ed. students	Direct	Qualitative
10. Šimić Šašić and Atlaga	Student perception of teacher encouragement of SRL	Examining how students perceive teacher encouragement of SRL and its association with their own SRL	School students	Indirect	Quantitative
11. Stephenson et al.	Helping teacher education students' understanding of SRL	Investigating the effect of SRL Teacher Promotion Framework (SRL-TPF), which focused on SRL promotion	Higher ed. students	Direct	Mixed-method
12. Sun et al.	Shyness and academic procrastination	Investigating the correlation between shyness and academic procrastination and the internal mechanisms	School students	Indirect	Quantitative
13. Wang et al.	Enhancing engagement through teacher expectations	Examining the longitudinal effect of students' perceptions of teacher expectations on their academic engagement	School students	N/a	Quantitative
14. Wolff et al.	Using the fused graphical lasso to explore the motivational self-system	Testing the effect of an intervention to improve SRL in biology courses using joint estimation of graphical models	Higher ed. students	Direct	Quantitative
15. Won and Chang	Antecedents and consequences of academic help-seeking in online STEM learning	Investigating the role of academic help-seeking in online STEM learning	Higher ed. students	N/a	Quantitative
16. López-Angulo et al.	Validation of the self-regulation of learning instrument for undergraduates	Designing and validating the SRLI-U scale that assesses SRL among Undergraduates	Higher ed. students	N/a	Quantitative
17. Zhao et al.	Variations in online SRL abilities	Understanding teachers' Online SRL abilities	Teachers	N/a	Quantitative
18. Zhu et al.	Fostering learning engagement	Examining the impact of relationships on engagement and the mediating role of SRL	School students	Indirect	Quantitative

## Teacher's perspective

Teachers play a key role in fostering SRL. While teachers universally recognize the importance of SRL, research reveals a complex landscape of implementation challenges and contextual variations.

The studies in this section reveal that teachers' fostering practices vary between direct and indirect approaches or lack clarity about both. Understanding this distinction helps explain the contradictions in findings. Several studies point to concerning gaps in teachers' SRL knowledge and implementation. Amir explored the innovative concept of “literacy resilience” as a key link between language literacy and SRL and sought to explore teachers' perceptions of its importance and their assessment of students' literacy resilience. Teachers emphasize the high importance of language literacy but perceive low levels of development in students, suggesting a gap between recognized importance and current development. She also found a significant lack of teachers' knowledge of literacy skills and SRL strategies. Similarly, the study by Manuel et al. aimed to explore the prevalent types and levels of feedback and how this feedback is used to promote the development of students' SRL skills. Participants predominantly used traditional, transmission-based teaching approaches, demonstrating limited understanding of various self-regulation skills that could improve academic achievement, particularly in native English speakers. Findings suggest a mismatch between teachers' perceptions of their feedback practices and the actual implementation of these practices in promoting students' SRL skills. These findings emphasize the gap between teachers' SRL knowledge and their practice.

However, these findings stand in contrast to other research showing teachers' confidence in SRL implementation. Greenquist-Marlett et al. found that teachers were generally confident in incorporating SRL into their teaching. They investigated how teachers from preschool to fifth grade apply SRL in their teaching, how their use of SRL strategies is related to their self-efficacy or confidence in teaching, and how teachers differ in their use of SRL depending on the type of school (public vs. private). Teachers often used SRL in implicit ways, setting goals based on students' needs, monitoring students' progress, and thereby adapting their teaching. Public school participants relied on time management and monitored student progress in more summative ways than their private school counterparts.

This apparent contradiction in findings suggests that teacher effectiveness in promoting SRL may depend heavily on contextual and individual characteristics. Zhao et al. investigated the characteristics of teachers in online SRL in the dual role of student and educator. Data analysis revealed uneven development in different dimensions of online SRL among teachers, higher levels of self-efficacy and motivation of secondary school teachers compared to preschool and primary school teachers, higher levels of self-efficacy in teachers from urban areas, positive correlation of teacher self-efficacy with educational qualifications, and negative correlation with years of service.

The learning environment itself may also play a critical role in shaping teacher practice. To understand the qualities of effective learning environments for this aim, in a study by Barz et al., the researchers analysed pre-service teachers' SRL profiles in asynchronous and synchronous digital learning environments, and compared their influence on training effectiveness. Their findings emphasize a person-centered approach to developing digital learning environments.

Beyond individual teacher characteristics and learning environments, interpersonal dynamics emerge as crucial mechanisms. Zhu et al. examined the influence of parent-child, teacher-student, and peer relationships on learning engagement and the mediating role of intentional self-regulation. The findings reveal that parent-child, teacher-student, and peer relationships have similar significant positive effects on learning engagement in middle school students. Intentional self-regulation has a partial mediating effect between all three types of relationships and learning engagement, with the strongest effect being found in teacher-student relationships. However, the unique effect of peer and parent relationships on learning engagement is significantly greater than that of teacher-student relationships.

It is necessary to identify significant contextual determinants of students' SRL and further differentially examine which specific variables of SRL these determinants have an impact on. With their study, de Ruig et al. addressed teacher self-efficacy as well as teacher-student interactions as two potential contextual predictors that might foster students' metacognitive, motivational, and behavioral self-regulatory skills. Their findings suggest that teacher self-efficacy plays a particularly crucial role in the development of students' SRL.

Since teachers play a key role in teaching SRL to their students, it is imperative to provide teachers with SRL knowledge and practice already at the training stage.

## Training effectiveness

All the papers presented above recognized the need for targeted interventions and professional development for teachers. Stephenson et al.'s research investigated the details and effects of a short online Professional Learning Program designed to develop teacher education students'knowledge about promoting SRL in the classroom. The results showed that by the end of the program, over 85% of the participants could provide teacher instructions that included explicit SRL promotion and/or promoted students' SRL knowledge. Backers and Van Keer explored the roles, responsibilities, and challenges faced by coaches in running professionalisation programs focused on SRL. They identified four roles for process coaches: trainer, expert, coordinator, and learner. In these roles, process coaches experienced different challenges and tensions; for example, they faced challenges in defining their role as content experts or group learning facilitators. The professional development programmes examined here primarily focus on direct approaches, explicitly teaching teachers how to promote SRL strategies, though coaches also consider indirect environmental factors.

## Student perspective

The results of these studies contribute to the understanding of fostering SRL from a student's perspective and highlight the importance of student evaluations of fostering SRL. They also provide guidance to teachers on how to organise teaching with the aim of encouraging SRL. From the student viewpoint, both direct interventions (e.g., peer assessment) and indirect environmental factors (relationships, teacher expectations, sense of belonging) emerge as significant influences on SRL development.

Research demonstrates that students' perceptions of their teachers play a crucial role in their self-regulatory development. Šimić Šašić and Atlaga examined how students perceive the teacher's encouragement of SRL and its connection with their own SRL. Students perceive that teachers moderately to relatively highly encourage SRL. Student perception of the teacher's encouragement of SRL is a significant predictor of student SRL. Proactive SRL strategies (focus on adoption, elaboration, and goal setting) are explained to a much greater extent. Extending this understanding of teacher influence, the study by Wang et al. examines the longitudinal effect of middle school students' perceptions of teacher expectations on their academic engagement, as well as the mediating role of intentional self-regulation in this dynamic. The results showed that students' perception of teacher expectations significantly predicted their academic engagement, with higher perceived teacher expectations leading to increased academic engagement. Furthermore, the study revealed that intentional self-regulation played a pivotal mediating role in the relationship between students' perceptions of teacher expectations and academic engagement.

These findings underscore the need to identify which specific factors contribute most to students' SRL development. A causal model of SRL can provide insight into what factors contribute to SRL, thus directing teachers to place their efforts in the most important factors. In a study by Kumyoung et al. examining a causal model of SRL, it was found that self-efficacy, achievement motive, and learning by imitation variables had a favorable impact on SRL. The researchers inferred that increasing self-efficacy, achievement motive, and learning by imitation among students may be an effective strategy for enhancing the efficiency of SRL. This research suggests that teachers should organize teaching and learning activities that promote achievement motivation and develop self-efficacy.

Recognizing that social factors like learning by imitation contribute to SRL, research has examined specific social learning strategies. Won and Chang investigated the role of academic help seeking, which is recognized as a learning strategy by SRL models, in online STEM learning and its contextual antecedents. Findings indicate that academic help-seeking is related to successful online STEM learning. This study also revealed a pathway through which sense of belonging and environmental fixed mindset are related to students' choice, retention intentions, and academic performance in STEM fields. Beyond help-seeking, peer interactions offer another avenue for developing self-regulatory competencies. Researchers have developed different approaches to promote SRL. Mostly, self-regulatory skills are fostered directly by offering training programs to the learners or to the teaching personnel. However, less is known about more indirect ways of fostering self-regulatory competencies. Thus, Ortega-Ruipérez and Correa-Gorospe conducted a systematic review in order to examine the effects of peer assessment in virtual classrooms of higher education on university students' metacognition and critical thinking. They found that peer assessment appears to be an effective and practical way to promote students' metacognitive skills.

While social strategies can support SRL development, individual characteristics may present obstacles that require attention. A study by Sun et al. examined the influence of shyness on academic procrastination and the role of self-regulation and self-directed attention in their relationship. They found that shyness significantly predicted academic procrastination, self-regulation mediated the relationship between shyness and academic procrastination, and higher levels of self-directed attention strengthened the predictive effect of shyness on self-regulation.

Given this understanding of contributing factors, barriers, and strategies, researchers have begun testing targeted interventions to enhance SRL. Wolff et al. investigated the effects of a randomized controlled trial designed to test the effect of a brief intervention used to improve SRL in an entry-level biology course using conjoint assessment of graphical models. The network structures of the experimental and control groups' motivational variables showed a high level of concordance in the relative magnitudes of edge weights; however, there were non-trivial differences in edge weights between groups that could be attributed to treatment and differences in predictability.

## Validation of measuring instruments

SRL skills are crucial for academic success at all levels of education. It is important to use valid and reliable measurement instruments to allow evaluating, monitoring, and intervening in SRL. For this purpose, in a study by López-Angulo et al., the researchers designed and validated the SRLI-U scale that assesses SRL among undergraduates, based on Zimmerman's model. Evans et al. also developed a scale to be implemented in the higher education context. However, their instrument does not address students; it rather aims at measuring academics' understanding of and training needs in assessment and feedback practices that focus on the systematic development of students' self-regulatory learning skills.

## Conclusions

This Research Topic makes a distinctive contribution to the field by focusing specifically on the fostering of SRL, shifting the research lens from examining how SRL affects academic success or how students perform self-regulation, to investigating how teachers, schools, and educational systems can actively promote and develop SRL competencies. This emphasis on fostering mechanisms represents a crucial but underexplored dimension in SRL research. The research presented in this Research Topic draws on contemporary theories and models of SRL (according to Panadero, 2017) and models of teacher facilitation of SRL (Karlen et al., 2020; Peeters et al., 2015; Kramarski and Heaysman, 2021). Research conducted with teacher samples still indicates that teachers recognize the importance of fostering SRL, but they lack the necessary knowledge and insufficiently foster SRL, most often using implicit strategies. Teacher self-efficacy is shown to be a significant predictor of encouraging SRL. Interpersonal relationships also play an important role in fostering SRL, as do feedback and teacher expectations that influence student engagement in learning. To better create a supportive synergy for enhancing students' learning engagement, families and schools need to provide consistent learning support. Research systematically reports on the effectiveness of teacher training in the area of developing and promoting SRL strategies. The results of the research presented in this Research Topic also indicate that special attention should be paid to teachers at lower levels of education (preschool and primary school levels), teachers from rural areas, and teachers with more years of experience. The results also contribute to understanding the promotion of SRL from the perspective of students and highlight the importance of student evaluations of the promotion of SRL. They also emphasize seeking academic help, learning by imitation, and peer assessment.

Interpreting findings through Dignath and Veenman's (2021) framework of direct vs. indirect fostering approaches reveals important patterns. Teacher's Perspective studies predominantly identified gaps in direct strategy instruction, while teachers showed more confidence in indirect methods such as goal-setting and progress monitoring. Training Effectiveness interventions focused primarily on equipping teachers with direct instructional strategies. Student Perspective research, however, highlighted the power of indirect approaches: relationships, teacher expectations, and peer assessment structures—suggesting these environmental factors may be underutilized. This pattern indicates that while research and training emphasize direct methods, indirect approaches warrant greater attention and systematic investigation.

### Directions for future research

While this Research Topic offers valuable insights, several important gaps remain that warrant attention in future research. First, the published papers predominantly focus on quantitative research methodologies. There is a clear need for more qualitative and mixed methods studies, especially when investigating research questions that have not been extensively explored yet. Emerging areas such as the intersection of SRL and artificial intelligence, for example, would benefit from in-depth qualitative investigations that can capture the complexity and nuances of these novel contexts. Second, in terms of generalizability, it is necessary to conduct more intercultural comparisons. The studies in this Research Topic included relatively few cross-country investigations with regard to their samples, limiting our understanding of how cultural contexts shape SRL promotion and development. Third, despite the demonstrated importance of teacher competencies in fostering SRL, there remains insufficient research on professional development programmes for teachers, particularly regarding the long-term effectiveness and sustainability of such interventions.

Papers within this Research Topic offer a synthesis of the latest knowledge in this research area, contribute to the consolidation of current knowledge, highlight limitations and critical issues of current research, and offer new ideas and thinking to support future research. We hope it will stimulate much future research.
